# Evaluating Expert-Layperson Agreement in Identifying Jargon Terms in Electronic Health Record Notes: Observational Study

**DOI:** 10.2196/49704

**Published:** 2024-10-15

**Authors:** John P Lalor, David A Levy, Harmon S Jordan, Wen Hu, Jenni Kim Smirnova, Hong Yu

**Affiliations:** 1 Department of Information Technology, Analytics, and Operations Mendoza College of Business University of Notre Dame Notre Dame, IN United States; 2 Center for Biomedical and Health Research in Data Sciences Miner School of Computer and Information Sciences University of Massachusetts Lowell Lowell, MA United States; 3 Tufts University School of Medicine Boston, MA United States; 4 Center for Healthcare Organization & Implementation Research Veterans Affairs Bedford Healthcare System Bedford, MA United States; 5 Precision for Medicine Flemington, NJ United States; 6 Manning College of Information and Computer Sciences University of Massachusetts Amherst Amherst, MA United States

**Keywords:** expert-layperson agreement, medical jargon, jargon identification, EHR, electronic health record notes, crowdsourcing, clinical notes

## Abstract

**Background:**

Studies have shown that patients have difficulty understanding medical jargon in electronic health record (EHR) notes, particularly patients with low health literacy. In creating the NoteAid dictionary of medical jargon for patients, a panel of medical experts selected terms they perceived as needing definitions for patients.

**Objective:**

This study aims to determine whether experts and laypeople agree on what constitutes medical jargon.

**Methods:**

Using an observational study design, we compared the ability of medical experts and laypeople to identify medical jargon in EHR notes. The laypeople were recruited from Amazon Mechanical Turk. Participants were shown 20 sentences from EHR notes, which contained 325 potential jargon terms as identified by the medical experts. We collected demographic information about the laypeople’s age, sex, race or ethnicity, education, native language, and health literacy. Health literacy was measured with the Single Item Literacy Screener. Our evaluation metrics were the proportion of terms rated as jargon, sensitivity, specificity, Fleiss κ for agreement among medical experts and among laypeople, and the Kendall rank correlation statistic between the medical experts and laypeople. We performed subgroup analyses by layperson characteristics. We fit a beta regression model with a logit link to examine the association between layperson characteristics and whether a term was classified as jargon.

**Results:**

The average proportion of terms identified as jargon by the medical experts was 59% (1150/1950, 95% CI 56.1%-61.8%), and the average proportion of terms identified as jargon by the laypeople overall was 25.6% (22,480/87,750, 95% CI 25%-26.2%). There was good agreement among medical experts (Fleiss κ=0.781, 95% CI 0.753-0.809) and fair agreement among laypeople (Fleiss κ=0.590, 95% CI 0.589-0.591). The beta regression model had a pseudo-*R*^2^ of 0.071, indicating that demographic characteristics explained very little of the variability in the proportion of terms identified as jargon by laypeople. Using laypeople’s identification of jargon as the gold standard, the medical experts had high sensitivity (91.7%, 95% CI 90.1%-93.3%) and specificity (88.2%, 95% CI 86%-90.5%) in identifying jargon terms.

**Conclusions:**

To ensure coverage of possible jargon terms, the medical experts were loose in selecting terms for inclusion. Fair agreement among laypersons shows that this is needed, as there is a variety of opinions among laypersons about what is considered jargon. We showed that medical experts could accurately identify jargon terms for annotation that would be useful for laypeople.

## Introduction

In recent years, federal regulations such as the Cures Act and the 2022 Office of the National Coordinator for Health Information’s Cures Act Final Rule [1] have mandated that health care providers grant patients full access to their electronic health records (EHRs) through patient portals [2]. Patient access to their EHRs represents a new communication channel between doctors and patients that can facilitate faster communication of test results, medication lists, and other information [3]. These regulations include the EHR notes as part of the data that must be provided [4]. Access to EHRs can enhance patients’ understanding of their disease [5], improve communication between patients and their care providers [6,7], improve medication adherence [8], and reduce health care costs [4,9-16]. However, EHRs contain medical jargon that patients, especially those with low health literacy [17], may not understand [18]. The following example is typical of the text that patients see in their EHR notes: “From a GI standpoint, we recommend to proceed with bariatric surgery. However, he will need to continue daily PPI administration to maximize acid reduction.” Without significant medical knowledge, it is challenging to understand the meanings of jargon terms such as “GI” (gastrointestinal), “bariatric,” and “PPI” (proton pump inhibitor).

Innovations are needed to support patients’ use of EHR notes and translate them into language that is easier for them to understand [19]. We found in our previous work that defining jargon and the readability of those definitions were positively associated with improved EHR note comprehension [20,21].

### Jargon in EHR Notes

According to the Cambridge Dictionary, jargon is defined as “language used by a particular group of people, especially in their work, and which most other people do not understand” [22]. Technical jargon occurs across disciplines and reflects the amount of specialist knowledge in a field [23]. Jargon can aid in communication by succinctly describing complex concepts. However, it can also impede communication with and comprehension by those unfamiliar with the language [24-26]. For accurate comprehension, a reader must be familiar with 95%-98% of the words in a text [27-30].

Medical records contain large amounts of jargon and abbreviations, and recent work has shown that patients cannot consistently comprehend the meaning of jargon terms, including abbreviations and acronyms [31-33]. Recent studies involving over 10,000 patients showed that allowing patients to read the clinical notes in their medical records confused some, especially those in vulnerable groups such as patients with lower literacy or lower income [4,34]. Similar work on patient portals shows that patients struggle to make use of their access [35-37].

There has been work in the literature on identifying and defining jargon in various fields, from medical informatics to scientific communication [23,30,38,39]. A significant gap in the previous work is that jargon identification is examined from an expert point of view. In medicine, consumer health vocabularies bridge the gap between patient and physician terminology. Consumer health vocabularies are typically collected by analyzing user search queries [40,41], web-based community forums [42-46], and patient-provider communications [41,47]. However, there is little work analyzing what patients recognize as jargon in the context of EHR notes. Furthermore, the literature has not examined if what patients recognize as jargon differs with demographic groups.

Recently, Pitt and Hendrickson [48] proposed a classification system for 7 types of medical jargon: technical terminology, alphabet soup, medical vernacular, medicalized English, unnecessary synonyms, euphemisms, and judgmental jargon. This categorization is broad enough to cover domain-specific technical terms (eg, “myocardial infarction” or “ambulating”) as well as more common words with specific medical meanings (eg, “progressing” or “positive”) [31,48].

### NoteAid

To assist patients with understanding medical notes, we are developing NoteAid [49], a natural language processing system that links medical jargon in EHR notes to definitions and addresses context-dependent meanings (eg, the abbreviation MAP could refer to muscle action potential or mean arterial pressure). A team of interdisciplinary experts identifies jargon from a corpus of EHRs and writes definitions for them. Specifically, NoteAid definitions of identified terms are written for a fourth- to seventh-grade reading level [50]. For example, in the EHR sentence referenced above, NoteAid would provide the following definition: bariatric surgery: Surgery on the stomach and intestines for weight loss. We have written definitions for approximately 30,000 jargon terms.

Our operational use of the term “medical jargon” in this study is patient-focused, not clinician-focused. NoteAid uses a 2-stage process for identifying jargon. First, the software generates a word frequency list from the EHR corpus. Starting from the most frequent word on the list, it presents sentences that contain the word to the definition writer. The definition writer reviews the sentence and decides which terms are jargon, following a set of guidelines in making this decision (more details in Multimedia Appendix 1). Examples of the guidelines include a medical term that would not be recognized by a layperson with a fourth-grade education (eg, duodenum); a word that has a different meaning in the medical context than in the lay context (eg, accommodate: when the eye changes focus from far to near). Still, the determination of what is and is not jargon is uncertain, as patients differ widely in their education, general level of health literacy, and experience with medical conditions.

In this observational study, we examined how well the NoteAid definition writers agreed with each other in identifying jargon and how well they agreed with laypeople. We used Amazon Mechanical Turk (MTurk) workers as proxies for laypeople. We also investigated how jargon identification varied across different demographic subgroups. We expected that demographic subgroups typically associated with lower health literacy would identify more terms as jargon than other subgroups. These groups include older adults, certain race or ethnic groups, those with a high school education or less, those whose native language is not English, and those who score low on a health literacy screener [51].

## Methods

We conducted an observational study to examine the agreement between the NoteAid definition writers and laypeople on what is considered medical jargon.

### Data Source

The NoteAid dictionary used medical notes from the PittEHR database of deidentified inpatient medical records [52]. The records consist of emergency department notes, progress notes, consult notes, operative reports, radiology reports, pathology reports, and discharge summaries, all written by physicians. We randomly selected 20 sentences from the database. Sentences that contained only administrative data, contained fewer than 10 words, or were substantially similar to another selected sentence were not included. The NoteAid definition writers had not previously seen these sentences.

### Identifying Terms for Annotation Task

The 20 sentences contained a total of 904 words. So as not to inflate the calculated agreement, we excluded from the analysis common words, which we defined as all conjunctions, pronouns, prepositions, numerals, articles, contractions, names of months, punctuation, and the 25 most common nouns, verbs, adjectives, and adverbs, including their plural forms. Terms that were repeated in a sentence were only counted once. Multiword terms were analyzed as single terms to avoid double counting. We considered multiword terms to be adjacent words that represented a distinct medical entity (examples include PR interval, internal capsule, and acute intermittent porphyria), terms that were routinely used together (examples include hemodynamically stable, status post, and past medical history), or terms that were modified by a minor word (examples include trace perihepatic fluid, mild mitral regurgitation, rare positive cells, and deep pelvis). The grouping of multiword terms was determined by 2 members of the research team after reaching a consensus. There were 325 potential jargon terms in the final analysis. We performed a second analysis in which only the common words were excluded, and there was no grouping of multiword terms. This process resulted in 549 potential jargon words in the analysis.

### Data Collection

Data collection took place between August 2020 and April 2021. NoteAid definition writers and MTurk workers were shown the 20 sentences. MTurk workers were asked to identify those terms for which they did not know the definition. In this paper, we refer to these identified terms as “jargon.” The NoteAid definition writers were asked to identify terms that they considered to be jargon, that is, terms for which laypeople would not know the definition. MTurk workers were instructed not to consult any sources of information during the task. Interspersed among the 20 sentences were 3 attention-check questions to test whether the participant was paying attention. If a participant answered 2 of the 3 attention checks incorrectly, the participant’s responses were discarded, and the participant was replaced (however, the participant was not excluded from reentering the study).

### Participant Recruitment

We recruited adult MTurk workers and collected demographic information about the workers’ age, sex, race or ethnicity, education, native language, and health literacy. We performed subgroup analyses based on MTurk worker characteristics. To evaluate health literacy, MTurk workers were screened with the Single Item Literacy Screener [51]. MTurk workers who worked in the health care field were excluded from the study. We also excluded MTurk workers with a previous approval rating below 95% in the MTurk platform.

MTurk workers were oversampled to obtain equal numbers of MTurk workers in each of the education subgroups and an equal number of non-White and White participants. We sampled 270 MTurk workers and 6 definition writers to complete the study instrument. The 6 definition writers were all experienced biomedical annotators with advanced degrees in medicine, nursing, biostatistics, and biomedical research.

### Evaluation

Our evaluation metrics were the proportion of terms rated as jargon, sensitivity, specificity, and Fleiss κ for agreement among NoteAid definition writers and among MTurk workers. Wald CIs were calculated at 95%. We analyzed NoteAid definition writers individually and as a group.

Sensitivity and specificity measured the NoteAid definition writers’ ability to correctly discriminate between jargon and nonjargon, using the MTurk workers’ responses as the gold standard. Since all 270 MTurk workers did not agree on which terms were jargon, the cutoff number of MTurk workers for defining a term as jargon was chosen using the Youden index [53]. Youden index calculates sensitivities and specificities for all possible thresholds (ie, between jargon and nonjargon). The cutoff where the sum of sensitivity and specificity was highest was selected.

To determine whether the definition writers’ jargon selection was systematically different from the MTurk workers, we calculated the Kendall rank correlation statistic between the definition writers and MTurk workers [54]. We also analyzed the results by MTurk worker characteristics to determine if specific subpopulations differed from the definition writers in the terms they identified as jargon.

We fit a beta regression model with a logit link to examine the association between MTurk worker characteristics and whether a term was classified as jargon. We used beta regression because it does not assume that individual terms have the same probability of being rated as jargon (eg, joint vs articulation). Here, the proportion of terms an MTurk worker identified as jargon served as the dependent variable, and the MTurk worker characteristics (sex, age group, race or ethnicity, education level, native language, and health literacy score) served as the independent variables. We assumed a linear relationship between the predictor and dependent variables, which we confirmed by checking the residuals. To check for the possibility of interactions among the independent variables, we explored adding different combinations of 2-way interaction terms. Models were evaluated using pseudo-*R*^2^ [55] and the Akaike information criterion (AIC) values [56].

### Ethical Considerations

We conducted this study with approval from the institutional review board at the University of Massachusetts Lowell (H00010621). Informed consent was obtained from MTurk workers, and they had the option to leave the task at any time. MTurk worker data were anonymized, and EHR data was deidentified. MTurk workers were paid US $3 for the task, which took an average of 20 minutes to complete.

## Results

Table 1 shows the characteristics of the 270 MTurk workers. The average proportion of terms identified as jargon by the MTurk workers overall was 25.6% (95% CI 25%-26.2%, Table 1). This proportion compares to 59% for the NoteAid definition writers (95% CI 56.1%-61.8%). Among MTurk worker subgroups, the average proportion of terms identified as jargon ranged from 17.7% to 30.9% (Table 1). Participants with the lowest health literacy score (5) identified fewer terms as jargon (*P*=.15, n=2), while participants with the second-lowest health literacy score (4) identified more terms as jargon (*P*=.03, n=10). Both sample sizes were small.

**Table 1:** Amazon Mechanical Turk worker demographics and the average proportion of terms identified as jargon.

Layperson characteristic			Values, n (%)		Proportion of terms identified as jargon^a^, %		95% CI (Wald)		
Overall			270 (100)		25.6		25-26.2		
**Sex**									
	Female	149 (55.2)		25.6		24.8-26.4			
	Male	120 (44.4)		25.6		24.8-26.5			
	Other	1 (0.4)		23.7		14.2-33.2			
**Age (years)**									
	18-24	16 (5.9)		25.4		23-27.7			
	25-34	74 (27.4)		25.6		24.5-26.7			
	35-44	65 (24.1)		24.9		23.7-26.1			
	45-54	27 (10)		26.2		24.4-28			
	55-64	59 (21.9)		25.4		24.2-26.6			
	65-74	27 (10)		27.4		25.6-29.1			
	75 and over	2 (0.7)		27.5		21-34.1			
**Education**									
	High school degree or less	90 (33.3)		25.3		24.3-26.3			
	Bachelor’s or associate’s degree	90 (33.3)		25.3		24.3-26.3			
	Master’s degree or higher	90 (33.3)		26.3		25.3-27.3			
**Race or ethnicity**									
	White	135 (50)		25.3		24.5-26.1			
	Asian	57 (21.1)		25.9		24.7-27.2			
	Black	46 (17)		25.8		24.4-27.2			
	Hispanic	15 (5.6)		27.3		24.9-29.7			
	Other	16 (5.9)		25		22.7-27.3			
**Native language**									
	English or bilingual	266 (98.5)		25.7		25.1-26.3			
	Non-English	4 (1.5)		21.6		16.8-26.4			
**Health literacy score^b^**									
	1 - Never	133 (49.3)		25.9		25.1-26.7			
	2 - Rarely	95 (35.2)		25		24.0-25.9			
	3 - Sometimes	30 (11.1)		25.3		23.6-27			
	4 - Often	10 (3.7)		30.9		28.1-33.8			
	5 - Always	2 (0.7)		17.7		10.7-24.7			

^a^Out of 325 potential jargon terms.

^b^Response to the question: “How often do you need to have someone help you when you read instructions, pamphlets, or other written material from your doctor or pharmacy?” [50].

### Model

The beta regression model had a pseudo-*R*^2^ of 0.071 (Table 2), indicating that the amount of variability in the proportion of terms identified as jargon explained by the MTurk worker characteristics was very low. The only significant MTurk worker characteristic was being a nonnative English speaker (*P*=.02; see Table 2). Compared with native English speakers, nonnative English speakers had a 0.535 odds ratio (95% CI 0.321-0.892) of identifying terms as jargon, controlling for sex, age, race or ethnicity, education level, and health literacy score. However, there were only 4 nonnative English speakers in the sample. Of the various 2-way interactions examined, only the addition of an interaction between race or ethnicity and education yielded a good model fit, and the interaction term was not statistically significant. The addition of this interaction slightly increased the proportion of variability explained (pseudo-*R*^2^=0.089) without meaningfully changing the AIC.

**Table 2:** Beta regression of proportion of Amazon Mechanical Turk workers marking terms as jargon.

Predictors			Participants, n	Coefficient	95% CI	*P* value
(Intercept)			—^a^	0.320	0.252-0.405	<.001
	**Sex**					
		Female (reference group)	149	—	—	—
		Male	120	0.985	0.883-1.099	.79
		Other	1	0.997	0.426-2.332	>.99
	**Age (years)**					
		18-24 (reference group)	16	—	—	—
		25-34	74	1.067	0.844-1.348	.59
		35-44	65	1.042	0.820-1.324	.74
		45-54	27	1.162	0.887-1.522	.28
		55-64	59	1.080	0.849-1.374	.53
		65-74	27	1.187	0.908-1.551	.21
		75 and older	2	1.262	0.684-2.325	.46
	**Education**					
		High school degree or less (reference group)	90	—	—	—
		Bachelor’s or associate’s degree	90	0.968	0.852-1.100	.62
		Master’s degree or higher	90	1.027	0.902-1.169	.69
	**Race or ethnicity**					
		White (reference group)	135	—	—	—
		Asian	57	1.07	0.933-1.226	.33
		Black	46	1.015	0.8781.174	.84
		Hispanic	15	1.157	0.922-1.451	.21
		Other	16	0.923	0.727-1.172	.51
	**Native language**					
		English or bilingual (reference group)	266	—	—	—
		Non-English	4	0.535	0.321-0.892	.02
	**Health literacy score^b^**					
		1 - Never (reference group)	133	—	—	—
		2 – Rarely	95	0.949	0.846-1.064	.37
		3 – Sometimes	30	1.002	0.845-1.188	.98
		4 – Often	10	1.252	0.963-1.627	.09
		5 – Always	2	0.571	0.294-1.113	.10
Observations			270	—	—	—
*R* ^2^			0.071	—	—	—

^a^Not applicable.

^b^Response to the question: “How often do you need to have someone help you when you read instructions, pamphlets, or other written material from your doctor or pharmacy?” [50].

### Agreement Among MTurk Workers and Among Definition Writers

The proportion of terms identified as jargon by NoteAid definition writers ranged from 48.3% to 64.9%. The agreement among NoteAid definition writers was good (Fleiss κ=0.781, 95% CI 0.753-0.809, Table 3), with all agreeing on the categorization (jargon or not jargon) for 74.5% of terms. The proportion of terms identified as jargon by individual MTurk workers ranged from 1.2% to 57.5%. Agreement among MTurk workers was fair (Fleiss κ=0.590, 95% CI 0.589-0.591, Table 3). For 61.9% of terms, at least 90% of MTurk workers agreed on the categorization (jargon or not jargon).

**Table 3:** Agreement among NoteAid definition writers and among Amazon Mechanical Turk workers.

	Participants, n	Fleiss κ	95% CI
NoteAid definition writers	6	0.781	0.753-0.809
MTurk workers	270	0.590	0.589-0.591

### Agreement Between Definition Writers and MTurk Workers

Our main measures of agreement between definition writers and MTurk workers were sensitivity and specificity. Using the Youden index, the highest combined sensitivity and specificity corresponded to at least 3 out of the 270 MTurk workers identifying a term as jargon. Using this cutoff, the mean sensitivity for the NoteAid definition writers was 91.7% (95% CI 90.1-93.3%), and the mean specificity was 88.2% (95% CI 86-90.5%; Table 4). These correspond to a false negative rate of 8.3% and a false positive rate of 11.8%, respectively. Among the individual NoteAid definition writers, sensitivity ranged from 78.1% to 95.8%, and specificity ranged from 79.7% to 94.7%.

Using the same threshold of 3 MTurk workers for classifying a term as jargon, we found that 59.1% of the terms would be classified as jargon, which is remarkably close to the average of 59% identified by definition writers.

The Kendall rank order correlation statistic was consistently high across all the MTurk worker characteristics, indicating no systematic differences in jargon identification between definition writers and different subpopulations of MTurk workers (Table 5).

All of the above analyses were repeated using single-word terms rather than multiword terms as the unit of analysis, and the results were not substantively different.

**Table 4:** Sensitivity and specificity of NoteAid definition writers.

Parameter	Value
Sensitivity^a^, % (95% CI)	91.7 (90.1-93.3)
Specificity^b^, % (95% CI)	88.2 (86.0-90.5)

^a^Sensitivity: Probability that definition writers identified a term that at least 3 MTurk workers had identified as jargon.

^b^Specificity: Probability that definition writers did not identify a term that less than 3 MTurk workers had identified as jargon.

**Table 5:** Kendall rank correlation coefficients comparing definition writer jargon ranks with Amazon Mechanical Turk worker jargon ranks.

Group			Participants, n		Kendall τ-b		
All MTurk workers			270		0.743		
**Sex**							
	Female	149		0.763			
	Male	120		0.762			
	Other	1		0.609			
**Age (years)**							
	18-24	16		0.754			
	25-34	74		0.768			
	35-44	65		0.758			
	45-54	27		0.759			
	55-64	59		0.754			
	65-74	27		0.759			
	75 and older	2		0.689			
**Education**							
	High school degree or less	90		0.759			
	Bachelor’s or associate’s degree	90		0.763			
	Master’s degree or higher	90		0.762			
**Race or ethnicity**							
	White	135		0.749			
	Asian	57		0.768			
	Black	46		0.772			
	Hispanic	15		0.782			
	Other	16		0.726			
**Health literacy score**							
	1	133		0.758			
	2	95		0.756			
	3	30		0.776			
	4	10		0.822			
	5	2		0.555			
**Primary language**							
	English	249		0.76			
	English, Arabic	1		0.753			
	English, Bengali	1		0.312			
	English, Chinese	8		0.757			
	English, Other	1		0.701			
	English, Spanish	5		0.746			
	English, Tamil	1		0.544			
	Other	1		0.704			
	Tagalog	2		0.713			
	Tamil	1		0.125			

## Discussion

### Principal Findings

Jargon identification depends on the target audience. Patients differ widely in their education, general level of health literacy, and experience with medical conditions [21,57]. What should or should not be considered jargon is not often clear, as evidenced by recent attempts to formalize the notion in a classification system [48]. Using sensitivity and specificity as measures of agreement, we found good agreement between definition writers and MTurk workers. The high level of sensitivity indicates that definition writers were providing definitions for the terms that laypeople identify as jargon. Correspondingly, the high specificity indicates that the definition writers would not be expending time writing definitions for terms that laypeople do not identify as jargon. These findings validate one goal of the NoteAid project, which is to assist patients in understanding their EHR notes, even if they have limited health literacy.

The calculation of sensitivity and specificity for the NoteAid definition writers required a gold standard as to which terms are and are not jargon. However, since all 270 MTurk workers did not agree on which terms were jargon, we used the Youden index to determine the cutoff number of MTurk workers for defining a word as jargon. In this method, sensitivities and specificities were calculated for all possible cutoffs, and the cutoff whose summed sensitivity and specificity were highest was used. This technique gave the best balance between sensitivity and specificity, though it treats the cost of false positives and false negatives as the same. On average, we found that MTurk workers identified 25.6% (22,480/87,750) of terms as jargon, compared with 59% (1150/1950) of terms identified as jargon by the definition writers. However, this is not necessarily undesirable. The definition writers were identifying jargon terms for inclusion in the NoteAid dictionary; broad coverage in terms of inclusion is preferable in this context. Further, MTurk workers differed considerably as to which terms they considered jargon (Fleiss κ=0.590), so simply matching their average proportion of terms identified as jargon would exclude terms that some laypersons consider to be jargon. Therefore, we also evaluated definition writers on their sensitivity and specificity in identifying MTurk workers’ jargon terms and found high agreement. These results suggest that personalized technologies such as NoteAid are needed, where specific results are identified from a wider database; a general consensus on what is or is not jargon may lead to the exclusion of terms that require definitions for a subset of the population.

Also, based on the MTurk worker health literacy scores, the average MTurk worker’s reading level was likely higher than fourth grade, the target level for NoteAid, which is consistent with previous work [21]. Therefore, the 25.6% jargon proportion among MTurk workers likely underestimates the prevalence of jargon terms for NoteAid’s target population.

Our beta regression model did not find differences between demographic subgroups in the proportion of terms identified as jargon. The only group that was significantly less likely to identify a term as jargon was the group for which English was not their native language. However, the small sample for this subgroup makes interpretation difficult. For the subgroups of adequate sample size, these results suggest that the selection of jargon by the NoteAid definition writers is sufficient.

In this work, MTurk workers and definition writers selected jargon terms from actual deidentified EHR notes. Most existing work looks at identifying jargon more generally, using data from web search logs or web forums. By using EHR notes for our task, the jargon identified should be more relevant for the downstream task of presenting definitions to patients looking at their own EHR notes.

### Limitations and Future Work

There are several limitations to this work that can inform future research. First, we examined a relatively small number of passages in this experiment. A different selection of passages could have produced different jargon identifications among the MTurk workers and definition writers.

A second limitation is that we did not examine context dependency in this study. For example, the term “tips” can be either nonjargon (suggestions) or jargon (transjugular intrahepatic portosystemic shunt). The NoteAid system considers context-dependent meanings, and future studies could address this.

Another limitation concerns our lay population. While MTurk is often used for crowdsourced data collection [58,59], the demographic characteristics of the collected sample are not representative of the broader US population [21]. In particular, we are interested in jargon identification behaviors for individuals with low health literacy, while the MTurk workers in our study generally had high health literacy scores. Of note is that the proportion of terms identified as jargon by MTurk workers in the lowest health literacy groups diverged from that of the higher health literacy groups. However, these sample sizes were very small, making interpretation difficult. Future work should attempt to replicate the results in actual patients, as in our previous work [21].

Using our demographic and other variables, the beta regression model only explained a small proportion of the variability in jargon identification among the MTurk workers. It is possible that there are unmeasured variables that would account for additional variability, such as income, occupation, personal experience with health issues, or interest in health topics.

The NoteAid definition-writing process is a distributed task. Each definition writer works on separate notes to identify and define jargon terms. Therefore, a consistent understanding of what is or is not jargon is important to ensure consistent coverage across the notes. Future development of NoteAid can investigate automatic jargon identification for definition writers through natural language processing tools, using the corpus of human-identified jargon as training data may lead to a more effective automated system if those data are consistent. In particular, with the growing impact of large language models [60-64], there is an opportunity to leverage large language models to improve patient understanding of notes. Future work can also use user information such as health literacy level and demographic information to identify the most relevant jargon terms and definitions, making the system even more personalized. Lastly, updating annotator instructions to be in line with established jargon classification frameworks can enforce consistency in labeling [48].

There are other applications for jargon identification, such as clinical trial regulations requiring plain language summaries [65]. This work can also inform jargon identification in other fields, such as law. A NoteAid-like tool for jargon identification and definition could define technical legal terms for lay individuals as they encounter them on the Web (eg, when reading a contract or terms of service agreement).

### Conclusion

In this work, we have shown that trained definition writers could consistently select jargon terms for which laypeople need definitions. These results are encouraging for the continued development of NoteAid, and they have implications for other fields.

## Appendix

Multimedia Appendix 1 NoteAid guidelines for identifying medical jargon.

## Abbreviations

AIC: Akaike information criterion
EHR: electronic health record
MTurk: Amazon Mechanical Turk

## Data Availability

The datasets generated during and/or analyzed during this study are available in a GitHub repository [66].
