# Supplementary material for: Evaluating Expert-Layperson Agreement in Identifying Jargon Terms in Electronic Health Record Notes: Observational Study
Source: J Med Internet Res. 2024 Oct 15;26:e49704. doi: 10.2196/49704 (PMC11522659; doi:10.2196/49704)
Supplement: Multimedia Appendix 1 [file jmir_v26i1e49704_app1.docx]

# Appendix A: NoteAid guidelines for identifying medical jargon

- Medical jargon is any medical term that would not be recognized by about a 5th grader, or that has a different meaning in the medical context than in the lay context, E.g.:

*accommodate: When the eye changes focus from far to near.*

*antagonize: A drug or substance that stops the action or effect of another substance.*

*resident: A doctor who has finished medical school and is receiving more training.*

*formed: Stool that is solid.*

- Terms that aren’t strictly medical, but are frequently used in medicine should also be defined. E.g.:

*aberrant*, *acute*, *ammonia*, *tender*, *intact*, *negative*, *evidence*

- While the focus is on medical jargon, non-medical jargon that is unfamiliar may also be defined. E.g.:

*PhD: [doctor of philosophy] A university degree given to people who become researchers.*

*MSG: [monosodium glutamate] A flavor enhancer in food.*

Terms whose definitions are widely known (e.g., by a 3rd grader) do NOT need to be defined. E.g.: *muscle*, *heart*, *pain*, *rib*, *hospital*

- When in doubt, define the term. E.g.:

*colon*, *immune system*

- When words are commonly used together, or together they mean something distinct or are difficult to quickly understand from the individual parts, define them. E.g.:

*vascular surgery: Medical specialty that performs surgery on blood vessels.*

*airway protection: Inserting a tube into the windpipe to keep it wide open and prevent vomit or other material from getting into the lungs.*

*posterior capsule: The thin layer of tissue behind the lens of the eye. It can become cloudy and blur vision.*

*right heart: The side of the heart that pumps blood from the body into the lungs.*

*intracerebral hemorrhage: A stroke.*

But NOT *right kidney, EMG findings, mildly advanced, intermittent distress, transection site, nonsurgical alternative, pericolic fat.* A guide to whether or not to capture a multi-word term is to ask yourself, “Would the definition for the multi-word term be superior to having separate definitions for each individual word?” E.g.,

*pleural effusion: Fluid build-up around the lung.*

vs.

*pleural: Relating to the lining around the lungs.*

*effusion: When fluid collects in hollow spaces or between tissues of the body.*
